# Effects of tongue right positioner use on tongue pressure: a pilot study

**DOI:** 10.1038/s41598-023-30450-0

**Published:** 2023-02-25

**Authors:** Ryosuke Yanagida, Koji Hara, Chizuru Namiki, Takuma Okumura, Akino Saiki, Kazuharu Nakagawa, Kohei Yamaguchi, Kanako Yoshimi, Ayako Nakane, Jean-Michel Mauclaire, Haruka Tohara

**Affiliations:** 1 Department of Dysphagia Rehabilitation, Graduate School of Medical and Dental Sciences, Tokyo Medical and Dental University (TMDU), Tokyo, Japan; 2 Department of Dentistry for the Special Patient, Kanagawa Dental University, 82 Inaokacho, Yokosuka, Kanagawa 238-8580 Japan; 3 Tongue Lab, Paris, France

**Keywords:** Medical research, Dentistry, Geriatrics

## Abstract

The effectiveness of the tongue right positioner (TRP) use on oral and swallowing functions remains unclear. To investigate the effects of TRP use on tongue function in patients with dysphagia. This interventional study included eight participants with dysphagia who visited a university dental hospital. The measurement variables included tongue pressure (TP) as the primary outcome and lip and tongue movements, peak nasal inspiratory flow, and changes in the tongue and suprahyoid muscle regions on ultrasonography as the secondary outcomes. Each participant was asked to use a TRP for at least 8 h every night for 2 months. The measurement variables before and after the intervention were compared using the paired *t* test and Wilcoxon signed-rank test. TP after intervention (31.5 ± 13.1 kPa) was significantly higher than that before intervention (23.0 ± 13.4 kPa), while other measurement variables did not significantly improve. Numerous exercises have been suggested to improve TP; however, most require patients’ adherence to instructions. In contrast, although participants did not perform active exercises, most participants in this study observed an improved TP. Our findings show that TRP can greatly improve TP after 2 months of usage.

*Trial registration number*: University Hospital Medical Information Network Clinical Trials Registry (UMIN000040253, date of first registration: 27/04/2020).

## Introduction

The tongue heavily interacts with the hard and soft palate during speech production as well as mastication and deglutition, cooperating with the lips, mandible, pharynx, and larynx. Therefore, tongue function impairment negatively influences speech production, masticatory, and swallowing movements, leading to dysphagia. Quantitative evaluation of the tongue function is essential for dysphagia rehabilitation, and tongue pressure (TP) is used as a quantitative and convenient index of tongue strength. However, decreased TP is associated with reduced swallowing and masticatory function^1,2^. TP can also be decreased by dysphagia-causing conditions, such as cerebrovascular diseases^3^, neuromuscular dysfunction^4^, Parkinson’s disease^5^, and sarcopenia^6^. In addition, recent evidence suggests that TP may also be decreased in patients with cognitive decline and mild cognitive impairment^7,8^. Therefore, a reduction in TP should be considered since it may serve as a valuable index for rehabilitation in dysphagia cases. Regarding the relationship between clinical symptoms and TP, prolonged mealtime, decrease in meal amount^9^, the occurrence of aspiration^10^, and pharyngeal residue^11^ have been reportedly associated with decreased TP. Consequently, it is necessary to improve TP during rehabilitation using exercises such as balloon tongue resistance^12^, tongue protrusion^13^, and tongue pressure resistance^14^ trainings.

**Figure 1:**
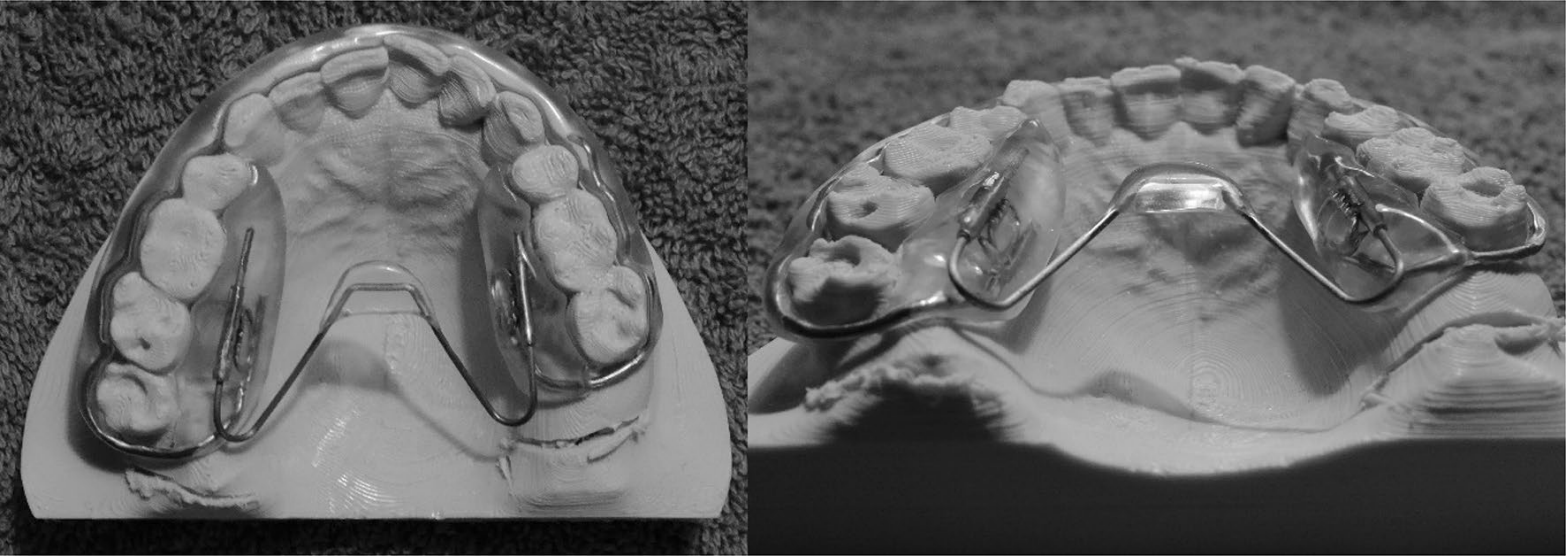
Views of the tongue right positioner. Left: Occlusal view; Right: Posterior view.

The tongue right positioner (TRP) (Fig. 1) is an oral device that was initially developed for orthodontic treatment^15^. Since TRP aligns the tongue’s position in the oral cavity, it is also used in patients with obstructive sleep apnea (OSA)^16^. The device partially inhibits the back and forward movements of the tongue. Therefore, it is believed that the strength of swallowing-related muscles increases to compensate for the inhibited tongue movements. A study reported that TRP increases the strength of suprahyoid muscles, such as geniohyoid and stylohyoid muscles^15^. Therefore, we hypothesized that TRP could potentially improve the strength of the tongue muscle. To the best of our knowledge, the effects of TRP on tongue function were investigated in patients with dysphagia for the first time.

## Methods

### Ethical considerations

This single-arm study was conducted per the Declaration of Helsinki of 1975, revised in 2013, and was approved by the Ethics Committee of the Tokyo Medical and Dental University (D2020-023). Written informed consent was obtained from all participants.

### Participants

Patients who visited a university dental hospital between September 2020 and September 2021 and had dysphagia were enrolled in this study. The inclusion criteria were as follows: (1) at least three of the six anterior teeth in the upper jaw remained, (2) at least two of the five molars on both the right and left sides of the upper jaw remained, (3) the lingual frenulum was not short, (4) those who could handle the use of the TRP, and (5) those who agreed to join this study. The exclusion criteria were the following: (1) inability to follow instructions, (2) altered consciousness, and (3) tracheostomy. Eight male patients with an average age of 58.8 ± 12.3 years received TRP intervention.

### Intervention

#### Tongue right positioner

The TRP (Tongue Lab Japan, Kyoto, Japan), patented by Tongue Lab., Paris, France, was provided to all participants. It is a custom-made removable oral device consisting of a resin button and a transverse arch (Fig. 1). The device was positioned along the maxillary arch, with the transverse arch extending from the first molar to the hard palate at the same height as the upper teeth. The resin button was located in the middle of the palate, and it opposed tongue movements. During the study, participants were asked to visit the hospital three times to receive the intervention. On the first visit, dental arch impressions were recorded to create a plaster model, which was sent to a dental laboratory to fabricate the personalized TRP. The participants receiving the TRPs were informed about their usage on the second visit, and they were asked to wear the device for at least 8 h every night while sleeping. Additionally, they were asked to return for a follow-up visit after 2 months to evaluate the effect of TRP use. Participants did not perform any active training during the interventional period.

#### Outcome

The outcome variables were assessed at the beginning of the study and at the 2-month follow-up visit. In this study, TP was the primary outcome, and the secondary outcomes were tongue and lip movement speed, peak nasal inspiratory flow (PNIF), and ultrasound assessment of swallowing-related muscles.

### Measurement

The measurements were performed by dentists who belong to the department of dysphagia rehabilitation in a university hospital and are accustomed to using the measurement devices. Before the measurements, the dentists calibrated the usage of all measurement devices employed in this study.

#### Tongue pressure

TP was evaluated using a JMS tongue pressure measurement device (JMS Co. Ltd., Hiroshima, Japan). Participants in the sitting position were asked to place the balloon in their mouth and hold the plastic pipe with their upper and lower central incisors with their lips closed. A dentist held the probe in the correct position while recording the measurements. The participants were then asked to push the balloon with their tongue against their hard palate for 7 s with maximum pressure. The TP was measured three times, and the average value was recorded as described previously^17,18^.

#### Tongue and lip movement speed

The tongue and lip movements were evaluated using oral diadochokinesis (ODK). ODK was measured using the KENKO-KUN Handy, an oral function-measuring device (Takei Scientific Co., Ltd., Niigata, Japan). Participants were asked to pronounce a monosyllable as quickly as possible for 5 s. The device recorded the number of repetitions for each syllable and calculated the number of syllables produced per second. The monosyllables ‘‘pa,’’ ‘‘ta,’’ and ‘‘ka’’ were used to evaluate the ability of the lips, the tip of the tongue, and the posterior region of the tongue, respectively^19,20^.

#### Ultrasonographic assessment of swallowing-related muscles

The cross-sectional area of the geniohyoid muscle (CSA of the GH) and the thickness of the tongue were evaluated using an ultrasonic measuring device (SonoSite M-turbo, Fujifilm, Tokyo, Japan) in B mode^21,22^ to evaluate swallowing-related muscles. The geniohyoid muscle was selected to represent the suprahyoid muscles. Participants were asked to gently close their mouths while facing forward in the sitting position to evaluate the CSA of the GH. The probe was placed with ultrasonic gel at the midline of the mouth floor to cover the geniohyoid muscle (sagittal), and it adhered adequately to the skin without applying pressure to the tissue. The probe was placed perpendicular to the Frankfurt plane on a line connecting the first mandibular molars of the left and right, including the second premolar, to evaluate the thickness of the tongue. The probes were brought into close contact with the lower surface of the mandible covered with ultrasonic gel^22,23^. The transverse section of the tongue was depicted at rest, with the participant facing forward in the sitting position.

The intraclass correlation coefficients (ICC) (1,1) and (2,1) were calculated to evaluate the reliability of the examiner. ICC (1,1) 0.925 and (2,1) 0.966 is used for measuring the CSA of the GH, and ICC (1,1) 0.936 and (2,1) 0.925 for the thickness of the tongue, which revealed high reliability. ImageJ software (National Institutes of Health, Bethesda, MD, USA) was used for image processing. The CSA of the GH and tongue thickness were measured thrice and twice, respectively, and the mean values were recorded. During the analysis, the examiner was blinded to the information, including the names of the participants and whether the image was taken at baseline or at follow-up.

#### Peak nasal inspiratory flow

A previous report^33^ on pediatric patients shows that TRP corrects the position of the tongue and improves upper airway patency. Therefore, we also measured PNIF in this study. The PNIF was evaluated using a portable PNIF meter (Clement Clarke International, Harlow, Essex, UK). Participants who held their heads parallel to the floor in a seated position were asked to inhale with their mouths closed as firmly and quickly as possible through the mask, starting from the end of full expansion. The reliability has been established previously^24,25^, with a correlation coefficient of approximately 92%^26^. The measurement was performed three times, and the average value was recorded as described previously^27^.

#### Other measurements

The Barthel index (BI), the functional oral intake scale (FOIS) scores, and the dysphagia severity scale (DSS) of the participants were recorded. The BI is an index of daily living activities consisting of 10 questions, with scores between 0 and 100. A higher score was associated with a higher physical function^28^. The FOIS, which is a seven-point scale, was recorded to assess the oral intake level, and a higher point was associated with a higher intake level. DSS is a seven-point scale, with scores of 1, 2, 3, 4, 5, 6, and 7 indicating saliva aspiration, food aspiration, water aspiration, occasional aspiration, oral problems, minimal problems, and within normal limits and no symptoms of dysphagia, respectively. The reliability of the BI, FOIS, and DSS has been verified previously^6,29–31^. At the 1- and 2-month follow-up visits, in addition to the measurement, the participants were asked whether they wore TRP every night, and if they did not, the number of times they forgot.

### Statistical analysis

The Shapiro–Wilk test was used to test the normality of all data, after which the paired *t* test and Wilcoxon signed-rank test were used for the analysis of parametric and non-parametric data, respectively, using the Japanese version of SPSS for Windows (version 25 J; IBM Japan, Ltd., Tokyo, Japan). Significant differences were considered at a corrected *p* < 0.05. A post hoc analysis was performed to calculate the effect size (ES) of each variable using G*Power 3.1 (Kiel University, Kiel, Germany). ES was defined as large for r > 0.5, medium for 0.3 < r < 0.5, small for 0.1 < r < 0.3, and without effect for r < 0.1.

### Ethics approval

This single-arm study was conducted per the Declaration of Helsinki of 1964, revised in 2013, and was approved by the Ethics Committee of the Tokyo Medical and Dental University (D2020-023).

### Consent to participate

Written informed consent was obtained from all participants.

## Results

**Figure 2:**
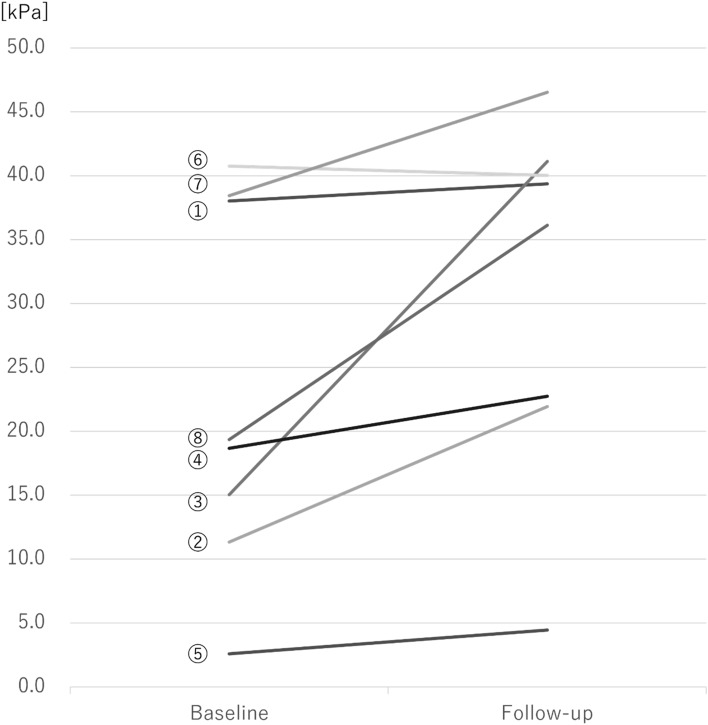
Comparison of tongue pressure between baseline and follow-up values for each participant. Each number indicates the participant’s number in Table 1.

**Figure 3:**
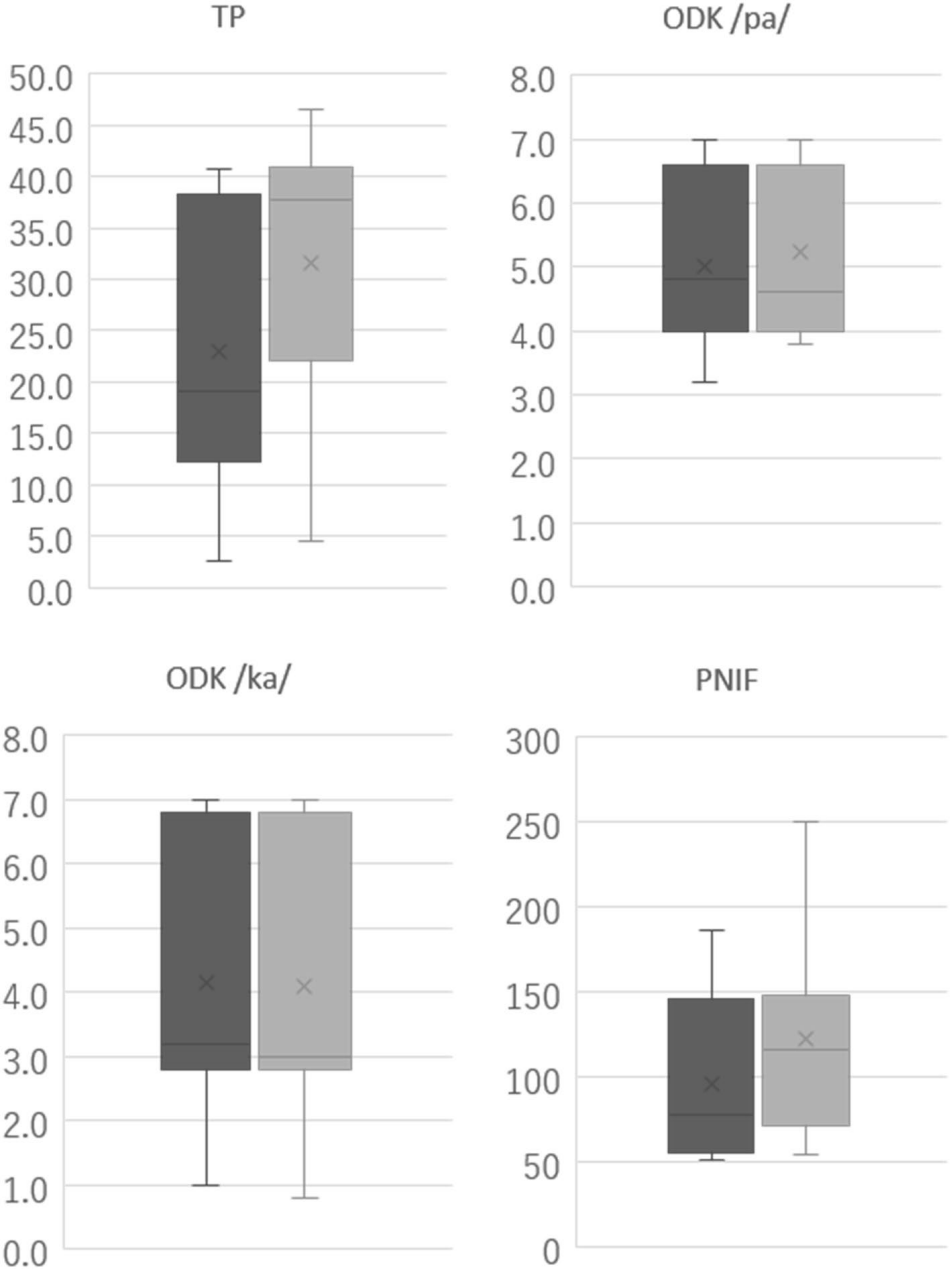
Box plots of measurement items with medium or large effect sizes. The left (dark grey) and right (light grey) box plots in each measurement item reveal baseline and follow-up values, respectively. The top and bottom of the vertical line show the maximum and minimum values, respectively, and the box in the middle illustrates the interquartile range. The horizontal line in the middle of the box shows the median value, and the cross mark shows the mean value. *TP* tongue pressure, *ODK* oral diadochokinesis, *PNIF* peak nasal inspiratory flow.

Eight participants (all men, average age of 58.8 ± 12.3 years) were enrolled in this study. Table 1 shows the characteristics of the participants. Dysphagia-causing diseases included neuromuscular diseases (n = 3) and cerebrovascular diseases (n = 2). The participants’ median BI, FOIS, and DSS scores were 85 (25–100), 6.5 (2–7), and 3.5 (2–4), respectively. Table 2 shows the differences in TP, ODK, PNIF, and ultrasonographic assessment of swallowing-related muscles before and after TRP use. A significant improvement was found in TP (*p* = 0.034, r = 0.84). The ES of each measurement item was also calculated. Of the eight participants, the ODK of one participant with cerebral infarction could not be evaluated because of expressive aphasia after cerebral infarction. There were no other missing data. All eight participants wore TRP every night during the 2-month intervention period. Figure 2 shows a comparison of TP between the baseline and follow-up values of each participant. Of the eight participants, seven showed improvements in TP. Figure 3 shows box plots of the measurement items with medium or large ES.

**Table 1:** Characteristics of participants.

No	Age	Sex	Weight	BMI	BI	FOIS	DSS	TP at baseline	TP at follow-up	Past history
1	56	M	106	35.8	100	7	4	38.0	39.4	Cerebral tumor
2	72	M	44	17.2	35	3	2	11.3	21.9	Progressive supranuclear palsy
3	49	M	78	26.7	70	6	3	15.0	41.1	Spinocerebellar degeneration
4	62	M	65	26.4	45	5	3	18.7	22.7	Amyotrophic lateral sclerosis
5	62	M	68	24.1	25	2	2	2.6	4.5	Cerebral infarction
6	67	M	59	22.5	100	7	4	40.8	40.0	Subjective symptoms of swallowing difficulty
7	70	M	71	28.4	100	7	4	38.4	46.5	Wallenberg syndrome
8	32	M	71	21.2	100	7	4	19.4	36.1	Subjective symptoms of swallowing difficulty

*BMI* body mass index, *BI* Barthel index, *FOIS* functional oral intake scale, *DSS* dysphagia severity scale, *TP* tongue pressure.

**Table 2:** Comparison of each measurement item between baseline and follow-up values.

Measurements (n = 8)		Baseline		Follow-up		*P* value	Effect size (r)
		Mean ± S.D	Median (interquartile range)	Mean ± S.D	Median (interquartile range)		
TP^a^	23.0 ± 13.4	19.0 (14.1–38.1)	31.5 ± 13.1	37.8 (22.5–40.3)	0.034*	0.84	
ODK	/pa/^b^	5.0 ± 1.3	4.8 (4.0–6.0)	5.2 ± 1.3	4.6 (4.0–6.6)	0.285	0.45
	/ta/^a^	5.0 ± 1.5	4.8 (3.6–6.6)	4.9 ± 1.4	4.6 (3.9–5.7)	0.668	0.27
	/ka/^a^	4.1 ± 2.1	3.2 (2.8–6.1)	4.1 ± 2.1	3.0 (2.8–6.1)	0.172	0.53
PNIF^a^	95.8 ± 48.4	78.3 (55.8–114.2)	122.1 ± 58.6	115.8 (73.8–147.1)	0.197	0.48	
Ultrasonographic assessment	CSA of GH^b^	174.9 ± 14.7	178.8 (175.1–182.6)	181.2 ± 24.0	188.0 (174.4–194.5)	0.484	0.25
	Thickness of tongue^a^	30.5 ± 3.9	30.4 (28.5–33.7)	29.8 ± 3.4	30.6 (28.2–33.7)	0.588	0.21

*S.D* standard deviation, *TP* tongue pressure, *ODK* oral diadochokinesis, *PNIF* peak nasal inspiratory flow, *CSA of GH* cross-sectional area of the geniohyoid muscle.

^a^Paired *t* test was performed for TP, /ta/, and /ka/ of ODK, PNIF, and the thickness of the tongue in ultrasonographic measurement.

^b^Wilcoxon signed-rank test was performed for /pa/ of ODK and CSA of GH in ultrasonographic measurement.

*Statistically significant (*p* < 0.05).

## Discussion

The TRP was employed for patients with dysphagia for the first time, showing that TP increased after 2-month use. Interestingly, although three participants had progressive disease, TP increased. Therefore, the device can serve as a valuable tool for functional rehabilitation of the tongue in patients with such diseases.

A decrease in TP was observed in only one of the eight participants. However, the TP of this participant at baseline was 40.8 kPa, which was not significantly low despite the presence of dysphagia, with a decrease of only 0.8 kPa. Therefore, TRP may not have been very useful in this case. However, TRP could be suitable for dysphagia caused by low TP.

Although a few mechanisms associated with TRP and improvement in TP have been reported, a possible explanation might be the lingual–hypoglossal reflex. When filiform papillae on the tongue are mechanically stimulated, the tongue’s tip curves upward, the tongue hollows from side to side with the upward curvature of the lateral edges, and the posterior part is depressed^32,33^. Consequently, TRP may cause this reflex and could enhance the strength of the tongue’s intrinsic and extrinsic muscles, which play a role in protrusion and retrusion. The improved tongue muscles could have increased TP, and TRP has been used to manage OSA^16^. The relationship between tongue protrusion strength in an arousal state and upper airway patency has been previously reported^34^. TP evaluation suggests that TRP improves the genioglossal muscle strength, contributing to tongue protrusion.

The ultrasonographic evaluation revealed that the CSA of the GH did not improve in this study (*p* = 0.484, r = 0.25). Although an association between TP and the geniohyoid muscle has been reported^15^, the mechanism of increased TP with TRP use could be owing to the enhanced tongue muscles rather than the suprahyoid muscles. However, it remains unclear whether TRP improved the geniohyoid muscle function in this study owing to the small sample size; therefore, additional research is required to clarify the effectiveness of TRP on the geniohyoid muscle function in a larger sample size. The thickness of the tongue did not improve (*p* = 0.588, r = 0.21) despite the improvement of TP. Tongue muscle strength increases without muscle hypertrophy at the beginning of muscular exercises because of neural adaptation^35^. The improvement in TP in this study can be attributed to this phenomenon.

The ODK and PNIF were also measured. The ‘Pa’ of ODK (r = 0.45) and PNIF (r = 0.48) during the follow-up visit tended to increase, but when compared with that of baseline, no significant differences were found. There may be a significant difference between baseline and follow-up values in future studies with a larger sample size. In this study, cases of some patients with progressive disease should have been adjusted or homogenized. Although the improvement was not significant, the PNIF scores increased. This finding is consistent with a previous study^36^. Similarly, the ‘Pa’ of ODK also increased. However, the reason behind this finding remains unclear. A possible explanation might be the reduction in mouth breathing caused by increased PNIF. Previously, adults’ mouth breathers had reduced nasal patency and lower PNIF scores than that of nasal breathers^37^. Particularly, mouth breathing is associated with weak lip muscle strength^38^. Therefore, TRP improved lip closure reflected in the ‘pa’ of ODK and PNIF. Moreover, the lip-closure strength should also be measured in future studies.

In contrast, the ‘ta’ and ‘ka’ of ODK decreased slightly. In the ‘ta’ of the ODK, the score of four participants, including the three participants with progressive neuromuscular diseases, increased whereas that of the other two decreased. The ‘ka’ scores of the two participants decreased, while no change was observed in that of the remaining participants. The improvement in the ‘ta’ could be explained by enhanced genioglossal muscle strength, similar to the improvement in TP. However, the ‘ka’ did not change or decrease. Of the two participants whose ‘ka’ decreased, one had progressive disease, and the other had a history of cerebral infarction. The ‘ta’ and ‘ka’ of ODK reflect the anterior and posterior movements of the tongue, respectively. TRP could maintain the movement of the anterior tongue despite the presence of progressive diseases.

This study had some limitations. First, only eight participants were included. Further, as we conducted this pilot study before conducting a randomized controlled trial with both TRP and placebo groups, we did not include a control group. Furthermore, confounding factors were not considered in this study. In the subsequent randomized controlled trial, the sample size will be set using the effect size calculated from the result of this study. Additionally, the participants were heterogeneous: the BIs, the FOIS scores, and the DSS ranged from 25 to 100, 2 to 7, and 2 to 4, respectively. Therefore, more studies are required with a larger number of male and female participants to clarify the effectiveness of TRP use in improving oral function. Second, although TP increased, the ultrasound assessment showed no improvement, suggesting that the muscle characteristics did not change after wearing TRP. Third, the increase in TP may be due to the lingual–hypoglossal reflex. However, it was difficult to prove that the reflex had actually occurred. Fourth, we only verbally confirmed the participants’ usage of TRP at 1- and 2-month follow-up visits. Although the compliance of the participants in this study was adequate that all of them wore TRP every night for the 2-month period, a checklist should be distributed to confirm the achievement rate in a future study. Fifth, we did not find any changes in DSS scores, evaluated by videoendoscope, between the initial and 2-month follow-up visits. The range of each DSS score is wide, and the DSS score does not reflect the movement of swallowing-related organs. Moreover, we could not evaluate the tongue’s position. Since the previous study indicated that TRP could improve the tongue’s position, leading to the increase of PNIF, we should have evaluated its position using videofluorography. Lastly, since the period of TRP usage was 2 months, TP possibly decreases after patients stop TRP usage. However, it remains unclear whether the effect of TRP continues even after the interventional period. Therefore, the long-term effect of TRP usage should also be evaluated in the future.

This is the first study to consider the oral or swallowing function of TRP users. Therefore, a randomized controlled trial should be conducted in future studies to emphasize the effectiveness of TRP in oral function. Considering that the ES of TP is 0.84 in this study, 48 participants, including 24 with TRP and placebo oral device (α = 0.05, power = 0.8), are required. Furthermore, since we did not observe any changes via the videoendoscope, swallowing evaluations with videofluorography should be conducted to observe the changes in swallowing kinematics and anatomical characteristics in future studies.

Numerous exercises have been suggested to improve TP^14,39,40^. However, most required patients to follow instructions. In contrast, although participants did not perform active exercises, most participants in this study observed an improvement in TP. In particular, participants Nos. 2 and 5 whose TP, BI, and swallowing function were low (Table 1) showed an increase in TP after 2 months of TRP use. This suggests that TRP can be useful for patients with dysphagia who cannot perform active exercises because of the low daily living activities. Additionally, TRP is a noninvasive and removable oral device, and caregiver assistance is required only during device insertion and removal. Therefore, TRP can be used even in situations with limited assistance. Furthermore, no adverse events, such as desorption, were reported in this study.

## Conclusion

This pilot study showed that TRP might improve TP in patients with dysphagia.

## Data Availability

The datasets generated during and/or analyzed during the current study are available from the corresponding author upon reasonable request.
